# Bioactive feed additives in animal nutrition: bridging innovation, health, and sustainability

**DOI:** 10.3389/fvets.2025.1727126

**Published:** 2025-12-18

**Authors:** Giovanni Buonaiuto, Tommaso Danese, Karim El-Sabrout, Arda Yıldırım

**Affiliations:** 1 Department of Veterinary Medical Science, Alma Mater Studiorum – University of Bologna, Bologna, Italy; 2 Department of Veterinary Science, University of Parma, Parma, Italy; 3 Department of Poultry Production, Alexandria University, Alexandria, Egypt; 4 Department of Animal Science, Faculty of Agriculture, Tokat Gaziosmanpaşa University, Tokat, Türkiye

**Keywords:** animal welfare, circular economy in feed resourches, dietary supplement, exogenous enzymes, functional feed ingredients, nutraceuticals, organic acids, phytogenics

## Abstract

Animal nutrition is shifting the focus from simply providing animals with feed to the nutrition of the whole system, balancing health, welfare, and ecological attention. This perspective synthesizes recent developments on bioactive feed additives (including phytogenic compounds, probiotics, prebiotics and synbiotics, exogenous enzymes, organic acid, and emerging options such as algal extracts, bioactive peptides, and fermented substrates) and evaluates their contributions to sustainable production. The aim of this paper is to outline how these interventions can enhance digestive efficiency, gut integrity, immune competence, and resilience to stress, thereby working with the aim to reduce antibiotic use, improve feed conversion, lower emissions, and valorization of agro-industrial by-products within circular economy schemes. Furthermore, appraising persistent bottlenecks will be covered, such as: heterogeneous responses across species and production contexts, narrow dose–response windows and interactions among multiple actives, limited evidence on long-term safety and carry-over into edible products, and fragmented regulatory pathways. Finally, a forward agenda will be proposed, which leverages multi-omics to elucidate host–microbe–diet mechanisms and define biomarkers of response; applies precision feeding and digital monitoring to individualize dosing; designs multifunctional formulations with complementary modes of action; and embeds One Health and life-cycle assessment to balance efficacy, safety, and sustainability. Reframed as strategic tools rather than ancillary supplements, bioactives can help build resilient, resource-efficient animal production systems.

## 1 Introduction

Animal nutrition is undergoing a remarkable transformation, moving from a discipline traditionally focused on the supply of essential nutrients, mainly for animal production and performance, toward a broader One Health concept, that encompasses animal health, welfare, and environmental sustainability (1, 2). Growing concern with antimicrobial resistance, added demand for safer and high-quality animal products, and the worldwide imperative to reduce the environmental footprint from agriculture and farming have collectively accelerated research on bioactive additives (2–4). While numerous studies have demonstrated their beneficial effects on feed efficiency, animal growth performance, and product quality, important knowledge gaps remain (1–7). Individual species-specific responses, definition of optimal dosages, the continued safety with prolonged usage, and the complexity of interactions with gut microbiota and host metabolism are still poorly understood (8, 9). In addition, the valorization of agro-industrial by-products as renewable sources of bioactive molecules presents both opportunities and regulatory challenges, requiring a careful balance between innovation and safety (7).

This perspective paper seeks to offer a holistic overview of recent advances in the use of bioactive feed additives and examine their potential as important facilitators of sustainable animal production highlight. Their active role in animal health and performance will also be highlighted, together with current tasks in addressing societal challenges, such as antimicrobial resistance and climate change, and the challenges that still need to be overcome to allow their full potential to be reached. Lastly, future directions will be outlined, including the use of omics technologies, precision feeding approached, and circular economy perspectives, which might be used to reposition bioactive additives from supplementary feed components to essential elements of resilient sustainable nutrition interventions.

## 2 Current advances in bioactive feed additives

**Figure 1:**
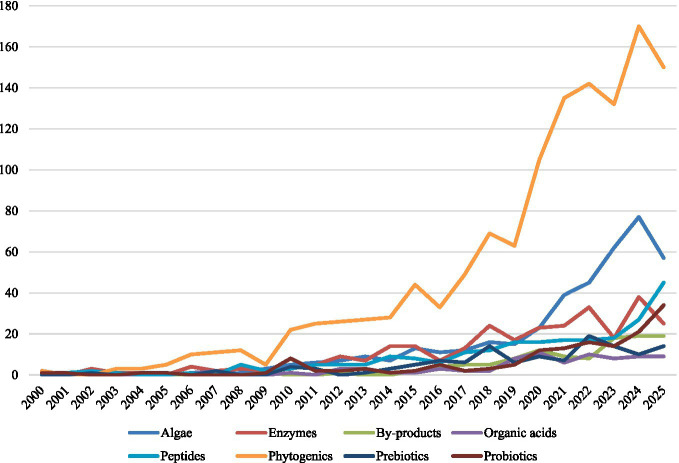
Temporal trends in scientific publications related to bioactive feed additives across additive categories (2000–2025)*. *Data were obtained from the Scopus database (Elsevier) using a bibliometric search conducted in September 2025. The query included the terms “feed additive,” “functional feed,” “nutraceutical,” or “bioactive,” combined with additive-specific keywords (e.g., probiotic, prebiotic, enzyme, phytogenic, organic acid, algae, peptide, fermented feed, by-product) within the Title, Abstract, or Keywords fields. Only articles referring to livestock, poultry, aquaculture, rabbits, equines, or companion animals were considered. The dataset was exported from Scopus and manually screened to classify publications according to the predominant additive type. The figure shows the annual number of publications per additive category.

During the last 20 years, the study of bioactive feed additives has gained significant traction (Figure 1), driven by the dual need to improve animal production and minimize reliance on antibiotic growth promoters (3). Bioactive feed additives are more than simply nutrients, but instead, they operate via a variety of actions including modulating gut microbiota, enhancing the activity of the host digestive enzyme systems, improving the absorption of nutrients, and regulating immune and oxidative responses (1, 6, 7, 10). Although their efficacy is influenced by multiple factors, namely as animal species, physiological stage, diet composition, and production environment, several classes of bioactive substances have resulted in reproducible and consistent results.

Phytogenics, or plant-derived compounds, represent one of the most widely studied categories (Figure 1). Essential oils, polyphenols, and other secondary metabolites are recognized for their antimicrobial, antioxidant, and anti-inflammatory activities (3). Recently, technological innovations have focused on stabilizing volatile phytochemicals utilizing encapsulated delivery systems, co-extrusion, and nano-delivery, which preserve their activity during feed processing and allow for a controlled-release application in the gastrointestinal tract. Such approaches have been useful in improving reproducibility and potency while preserving the stability of the gut microbiota and host antioxidant status (11, 12). By stabilizing the gut microbiome and controlling potential pathogenic overgrowth, phytogenics can improve digestive function and resilience to stress (13). Moreover, certain plant extracts have demonstrated immunomodulatory effects, enhanced disease resistance while maintaining growth performance (5, 14–16).

Probiotics and prebiotics are yet another cornerstone of bioactive nutrition (Figure 1). Probiotic strains such as *Lactobacillus*, *Bifidobacterium*, and *Bacillus* have been shown to be beneficial for gut health by competitive exclusion of pathogens, stimulating mucosal immunity, and producing beneficial metabolites (17). Prebiotics, such as inulin and oligosaccharides, can selectively stimulate beneficial microbial populations and enhance the production of short-chain fatty acids, thereby improving nutrient utilization and intestinal integrity (18). The concept of synbiotic (the combination of probiotics and prebiotics) has emerged as a new and exciting way to further enhance these synergistic effects (19).

Exogenous enzymes, such as carbohydrase, phytase, and protease, have been available for a long time to improve feed digestibility, but their role as bioactive additives is increasingly recognized (Figure 1). By breaking apart complex substrates, exogenous enzymes release nutrients otherwise unavailable to animal digestion reducing feed costs and nutrient excretion (20). Recent studies have expanded this application of exogenous enzymes to microalgae and other single-cell ingredients, where carbohydrate-active enzymes enhance the accessibility of proteins and lipids from complex cell matrices. This innovation not only improves digestibility but also optimizes the sustainable utilization of algae as a bioactive feed ingredient rich in antioxidants and PUFA (21). In addition, enzyme supplementation can interact synergistically with the gut microbiome, promoting a more favorable fermentation profile (20).

Another significant category is organic acids, an important component both in swine and poultry nutrition (Figure 1). Their antibacterial activity is largely attributed to their ability to penetrate bacterial cell walls and disrupt metabolism (22). Concurrently, organic acids decrease gastrointestinal pH, improving nutrient solubility and digestive enzyme activity (23). Beyond growth promotion, they have been linked to improved intestinal morphology and reduced incidence of enteric disorders (24). More recently, multi-component formulations combining organic acids with phytogenics or probiotic strains exhibit additive effects on growth, feed efficiency, and pathogen control. This expands the class of additives from simple acidifying agents to health-promoting, multi-functional additives (25).

Emerging bioactives such as algal extracts, peptides, and microbial metabolites are currently gaining traction (Figure 1). Algae contain novel bioactive molecules, such as polysaccharides, carotenoids, and polyunsaturated fatty acids, with demonstrated effects on immunity and oxidative balance (26). Bioactive peptides, derived from feed proteins or fermentation processes, are increasingly studied for their antimicrobial and immunoregulatory abilities (26). Fermented feedstuffs may also represent a source of bioactive compounds, and improve feed palatability, nutrient digestibility, and gut health (27). A schematic overview of the major classes of bioactive additives, their mechanistic axes, and representative outcomes is provided in Table 1.

**Table 1:** Conceptual mapping of major bioactive additive classes, mechanistic pathways, and sustainability-oriented outcomes.

Additive class	Representative examples	Primary mechanistic axes (conceptual)	Main functional outcomes (not exhaustive)
Phytogenics	Essential oils, plant extract	Microbiota modulation Antioxidant/redox axis Immune signaling Rumen fermentation effects	Antimicrobial activity Reduced oxidative stress Improved digestive efficiency CH₄ mitigation potential
Probiotics	Microrganisms	Microbial competition Mucosal immunity SCFA^b^/bacteriocin production	Pathogen exclusion Improved barrier function Better nutrient utilization
Prebiotics/synbiotics	Inulin; FOS^a^; MOS; synbiotic blends	Selective substrate SCFA^b^ formation Barrier integrity	Enriched beneficial taxa Improved gut morphology Synbiotic synergy
Exogenous enzymes	Carbohydrases; proteases; phytase	Substrate hydrolysis Nutrient release Fermentation coupling	Higher digestibility Reduced N/P excretion Improved FCR^c^
Organic acids	Formic; lactic; butyric acid	pH reduction Antimicrobial barrier Solubility improvement	Lower pathogen load Enhanced villi Improved enzyme activity
Emerging bioactives	Algal extracts; peptides; fermented substrates; postbiotics	Immune modulation Antimicrobial peptides Microbial signaling	Improved immune tone Anti-biofilm action Enhanced digestibility
Circular-economy bioactives	By-products; co-products	Polyphenol–fiber axis Waste valorization Microbial interactions	Lower feed cost Environmental benefits Functional compound source

aFOS, Fructooligosaccharides; MOS, Mannan-oligosaccharides.

bSCFA, Short-chain fatty acids.

cFCR, Feed conversion ratio.

## 3 Bioactive additives for sustainable animal production

The significance of bioactive feed additives goes beyond the direct effects on animal performance. These compounds are increasingly viewed as significant to the sustainability of livestock production systems. Their use is relevant to global priorities, aimed to reduce antibiotic usage, improving feed efficiency, decrease environmental emissions, and enhancing animal welfare, thereby responding to societal expectations and regulatory demands.

The need to reduce the dependence on antibiotic growth promoters has driven much of the acceptance of bioactive additives (28, 29). With the global ban or restriction of in-feed antibiotics, especially in Europe, it has been necessary to find alternatives to promote animal health and minimize the use of antibiotic growth promoters. Bioactives such as phytogenics, probiotics, and organic acids have demonstrated to lower the incidence of gastrointestinal infections and increase immune competence, thereby mitigating the need for prophylactic antibiotic use (30), and directly contributing to the global effort to mitigate antimicrobial resistance, one of the greatest public health concerns. Feed efficiency as an aspect of sustainability is also critical, as bioactive additives improve nutrient utilization by a variety of mechanisms; these include improved digestibility, modulation of gut microbiota, and stimulation of endogenous enzyme activity (1, 30, 31). By improving feed conversion ratios, bioactive compounds mean less feed is required to produce the same amount of animal product, encouraging the efficient use of resources and lowering production costs (5, 7). This improvement has important implications not only for farm profitability but also for reducing the environmental footprint of animal agriculture. In ruminants, some bioactive compounds have shown the potential to alter rumen fermentation patterns in ways that reduce methane emissions (32, 33). Tannins and saponins derived from plants, and some essential oils, have demonstrated efficacy in modulating methanogenic microbial populations, leading to lower greenhouse gas emissions without compromising animal performance (34, 35). This type of nutritional strategy supports broader climate change mitigation and recognizes the important role of nutrition in addressing environmental issues. Valorizing agro-industrial by-products and waste is another application that has value for both producers and the environment. Many agricultural residues, including oilseed cakes, fruit pomaces, cereal bran, and fermentation co-products, are rich in bioactive compounds such as polyphenols, fibers, and bioactive peptides (36, 37). Incorporating these by-products into livestock diets not only reduces feed costs but also promotes circular economy principles by transforming waste into value-added inputs (37). However, the use of such materials requires careful consideration of variability in chemical composition, potential antinutritional factors, and regulatory approval processes (38). Standardization, scalable supply chain and safety assessment remain major bottlenecks for widespread adoption.

Beyond performance and environmental outcomes, bioactive compounds contribute to animal health and welfare, a rightly increasingly component of sustainable production. By reducing oxidative stress, modulating immune responses, and supporting overall gut health, these additives help animals cope better with environmental and management-related stressors (37). Improved welfare not only benefits animals but also supports consumer acceptance, and therefore improves market value, of animal-derived products. However, realizing the sustainability potential of bioactive additives will require integrated life cycle and economic assessments that balance environmental benefits with energy demand, formulation costs, and efficacy variability across production systems (39).

## 4 Discussion and future perspectives

Despite considerable progress in the study and application of bioactive feed additives, several gaps continue to limit their full integration into modern livestock production. These limitations are scientific, practical, and regulatory in nature, reflecting the complexity of translating promising experimental products into consistent outcomes, viable under commercial conditions.

One initial challenge related to the variability of responses both across animal species and production stages. While some bioactives demonstrate consistent efficacy in controlled trials, their effects can be inconsistent when tested under field conditions (40). Variability in diet composition, housing, health, and genetic background greatly affects the outcomes, making it difficult to establish universal recommendations. Defining optimal dosages across species and delivery methods represents another critical issue (40). Many bioactive compounds have a relatively narrow effective dose, with suboptimal doses providing little to no measurable effects and excessive supplementation potentially impairing performance or leading to unwanted interactions. In addition, the combination of multiple bioactives (common in commercial feed formulations) may generate synergistic, additive, or antagonistic effects that are not yet fully understood. A more mechanistic understanding of dose–response relationships is required to optimize their application. Long-term safety and carry-over effects into animal-derived products also remain insufficiently studied. While short-term trials generally confirm the safety of most bioactives, fewer studies have evaluated the potential accumulation of bioactive metabolites in tissues or products such as milk, eggs, and meat (41). Consumer safety, product quality, and regulatory compliance depend on a robust assessment of these aspects, which must be integrated into future research agendas.

Another major challenge is the complexity of interactions between bioactives, the gut microbiota, and host immunity. Many bioactives exert their effects indirectly by shaping microbial communities and influencing microbial metabolites. However, the microbiome is highly dynamic and responsive to multiple factors beyond diet, which complicates the interpretation of results. Advanced omics technologies are beginning to unravel these interactions, but translating this knowledge into practical feeding recommendations remains a significant hurdle. Finally, regulatory frameworks present important barriers to innovation. Approval processes for new feed additives differ considerably across countries, and the classification of bioactives often falls into a gray zone between feed, feed additives, nutraceuticals, supplements, and veterinary products. This lack of harmonization increases the time and cost of bringing new products to market, discouraging innovation and limiting the global adoption of promising compounds.

One major opportunity is the integration of omics technologies (metagenomics, transcriptomics, metabolomics, lipidomics, and proteomics) to uncover the mechanisms through which bioactives exert their effects (42, 43). By providing a detailed understanding of host–microbe–diet interactions, these tools can identify biomarkers of response, predict efficacy in specific contexts, and guide the design of targeted interventions. Such knowledge will be critical for distinguishing between compounds with broad applicability and those requiring tailored use. Precision feeding and digital livestock farming represent another frontier. Advances in sensor technology, artificial intelligence, and data analytics make it possible to monitor animal health, behavior, and performance in real time (44). Linking this information to nutritional management opens the possibility of delivering bioactive additives in a dynamic, individualized manner, adjusting doses and combinations according to the animal’s physiological state, environmental stressors, or disease challenges. This shift could enhance both efficacy and cost-effectiveness.

The development of multifunctional feed additives also holds promise. Instead of relying on single compounds, future formulations may combine bioactives with complementary modes of action (such as phytogenics with probiotics, or enzymes with organic acids), creating synergistic effects on gut health, immunity, and nutrient utilization. Such approaches could provide more consistent results across production systems, though they will require rigorous testing to understand interactions and avoid redundancy. Sustainability imperatives will continue to drive innovation. The valorization of agro-industrial by-products rich in bioactives is expected to expand, offering not only a sustainable source of functional compounds but also a practical solution to reduce waste and improve resource efficiency (2, 45). Advances in biotechnology, such as fermentation and bioprocessing, will likely play a key role in enhancing the availability, consistency, and bioactivity of these materials. A broader One Health perspective will be essential in guiding future research and application (46). By improving animal health and welfare while reducing reliance on antibiotics and lowering environmental emissions, bioactive feed additives directly contribute to public health and ecosystem resilience. The next generation of studies should therefore adopt multidisciplinary approaches, integrating veterinary science, nutrition, microbiology, and environmental science. Finally, harmonization of regulatory frameworks will be critical to enable global innovation and adoption. Transparent, science-based approval systems that account for both safety and sustainability outcomes will facilitate the responsible use of bioactives. Collaboration among academia, industry, and policymakers will be necessary to establish these standards and ensure that innovation reaches commercial practice.

In conclusion, the future perspectives for bioactive feed additives are highly promising. Through advances in technology, interdisciplinary research, and regulatory alignment, these compounds can become central tools for achieving productive, resilient, and sustainable animal production systems. Bioactive feed additives are reshaping the landscape of animal nutrition, moving the discipline beyond the traditional provision of essential nutrients toward a more holistic approach that integrates health, welfare, and sustainability. Their proven effects on digestive efficiency, immune modulation, and stress resilience position them as valuable alternatives to antibiotic growth promoters and as key contributors to more resource-efficient production systems. At the same time, their ability to valorize agro-industrial by-products and reduce environmental emissions aligns with broader societal and policy goals addressing antimicrobial resistance, climate change, and circular economy principles. Nevertheless, the current body of evidence highlights several challenges that must be addressed to fully realize the potential of bioactives. Variability in animal responses, uncertainties around optimal dosages, and limited knowledge of long-term safety remain significant barriers. Equally important are the knowledge gaps concerning complex host–microbiome interactions and the lack of harmonized regulatory frameworks, which continue to slow innovation and adoption at the global level. Looking forward, advances in omics technologies, precision feeding, and digital monitoring tools will enable a more targeted and science-driven application of bioactives. Multifunctional formulations and the valorization of sustainable raw materials will expand their utility, while the integration of a One Health perspective will ensure benefits that extend beyond livestock production to human health and environmental resilience.

## Data Availability

The original contributions presented in the study are included in the article/supplementary material, further inquiries can be directed to the corresponding author.
